# Undifferentiated pleomorphic sarcoma in the anterior mediastinum with a rapidly progressive course: A case report

**DOI:** 10.17179/excli2020-2694

**Published:** 2020-08-17

**Authors:** Masamichi Sato, Sumito Inoue, Tsuyoshi Arao, Akira Igarashi, Keiko Yamauchi, Kento Sato, Hiroshi Nakano, Masafumi Watanabe

**Affiliations:** 1 Department of Cardiology, Pulmonology, and Nephrology, Yamagata University Faculty of Medicine, Yamagata, Japan; 2 Internal Medicine, Shiseido General Hospital, Yamagata, Japan

**Keywords:** mediastinal tumor, undifferentiated pleomorphic sarcoma, computed tomography-guided biopsy

## Abstract

A 77-year-old woman with heart failure was admitted to our hospital. Computed tomography (CT) of the chest revealed an anterior mediastinal tumor. CT-guided biopsy revealed a malignant nonepithelial tumor of unknown origin. She was not treated with chemotherapy or radiotherapy because of her poor clinical condition. She died 33 days after admission. Following autopsy, we confirmed that the mediastinal tumor had infiltrated the large blood vessels. After final histological examination, undifferentiated pleomorphic sarcoma was diagnosed. Primary mediastinal sarcomas are very rare; clinicians should be aware of their possibility because some cases may progress rapidly as evidenced in this case.

## Introduction

There are various histological types of mediastinal tumors; however, sarcoma is rarely seen in this location. Pleomorphic sarcoma is a soft tissue malignancy comprising several types of histologically undifferentiated atypical cells. Herein, we present an autopsy case of pleomorphic sarcoma that developed rapidly in the anterior mediastinum.

## Case Report

**Figure 1:**
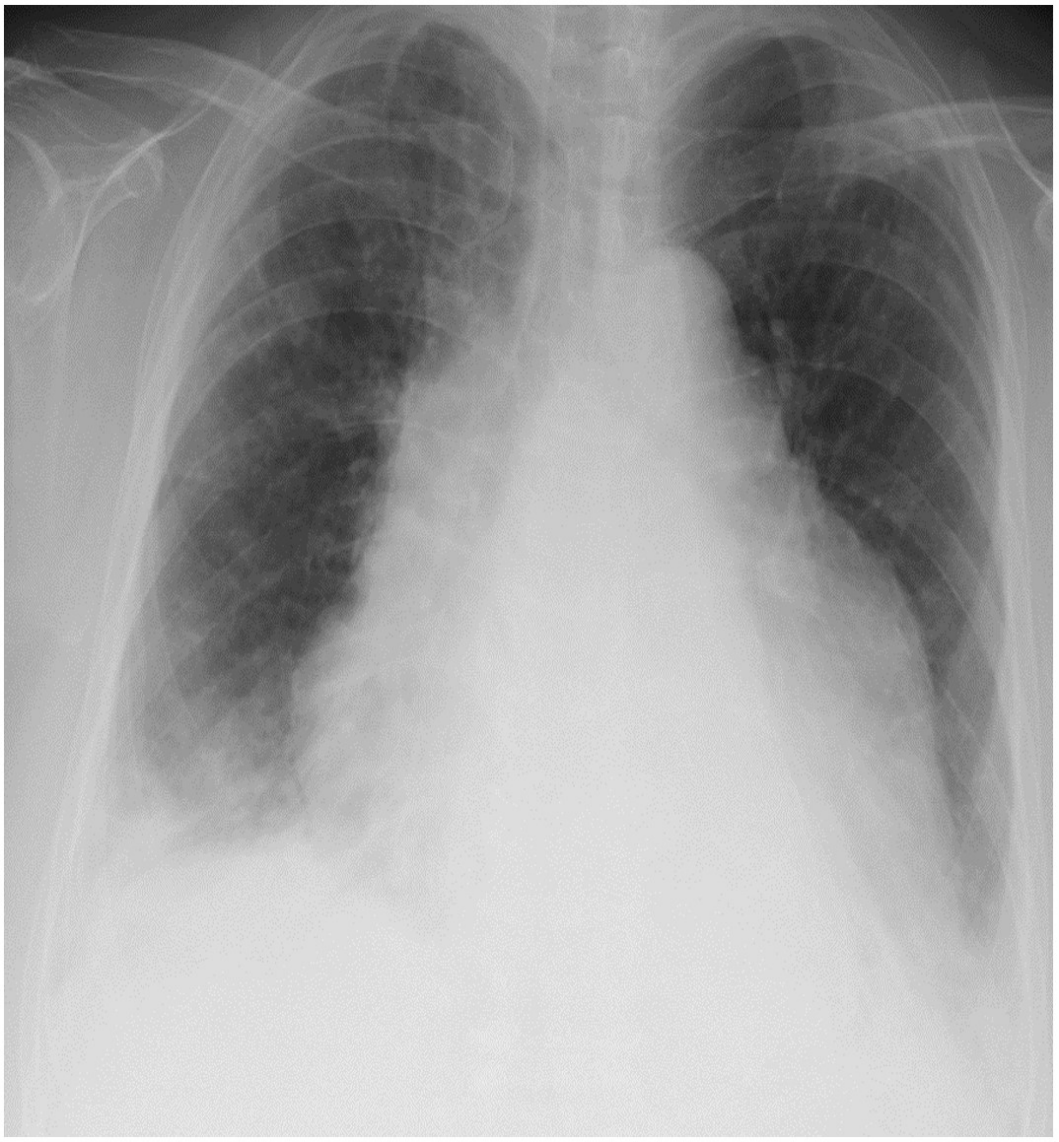
Chest radiography showed a cardiothoracic ratio of 66 % with cardiac enlargement and bilateral pleural effusions, which were greater on the right. Lung lesions were not clearly visible.

The patient was a 77-year-old woman who presented with a 1-month history of shortness of breath on exertion and palpitations. Her medical history was notable for diabetes, hypertension, hyperlipidemia, and subarachnoid hemorrhage. Her body mass index was 34.7 kg/m^2^. She was afebrile with blood pressure 109/71 mmHg, heart rate 92 beats/min, and SpO_2_ 96% on room air. Her physical examination was notable for bilateral lower extremity edema. No heart murmur nor abnormal breath sounds were auscultated. The conjunctiva, abdomen, and skin were normal. Neurological abnormalities were nor observed. Peripheral blood counts, including white blood cell fraction, were within normal limits. The lactate dehydrogenase (LDH) level was elevated (613 IU/L), and laboratory findings were consistent with mild generalized inflammation (C-reactive protein level was 1.45 mg/dL, and B-type natriuretic peptide level was 133.4 pg/mL). Human chorionic gonadotropin, alpha fetoprotein, carcinoembryonic antigen, squamous cell carcinoma antigen, and anti-acetylcholine receptor antibody levels were within the normal range. Chest radiography (Figure 1(Fig. 1)) showed a cardiothoracic ratio of 66 % with cardiac enlargement and bilateral pleural effusions. Lung lesions were not clearly visible. She was subsequently admitted to the hospital with a diagnosis of heart failure.

**Figure 2:**
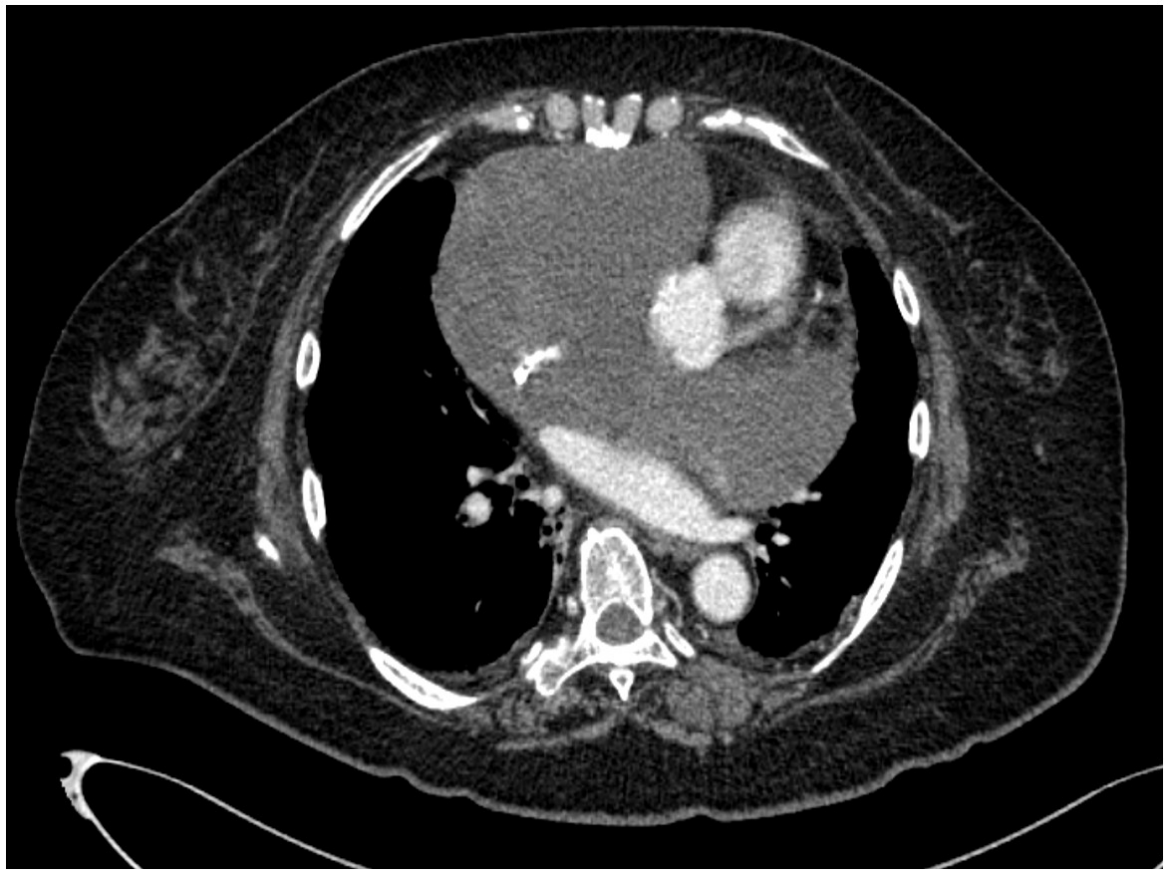
Contrast-enhanced chest computed tomography scan confirmed the ultrasound findings and showed mass effect on the superior vena cava, which was significantly narrowed. The inside of the tumor could not be clearly visualized on contrast-enhanced imaging; neither fatty nor calcified components were observed.

Electrocardiography revealed a heart rate of 90 beats/min, normal sinus rhythm, and mild ST depression in leads V3-V6. Transthoracic cardiac ultrasonography revealed a mass, which extended into the side of the right atrium, around the ascending aorta. There was a hypoechoic area within the mass, suggesting presence of fluid. Contrast-enhanced chest Computed tomography (CT) scan (Figure 2(Fig. 2)) confirmed the ultrasound findings and showed mass effect on the superior vena cava, which was significantly narrowed. The inside of the tumor could not be clearly visualized on contrast-enhanced imaging; neither fatty nor calcified components were observed.

**Figure 3:**
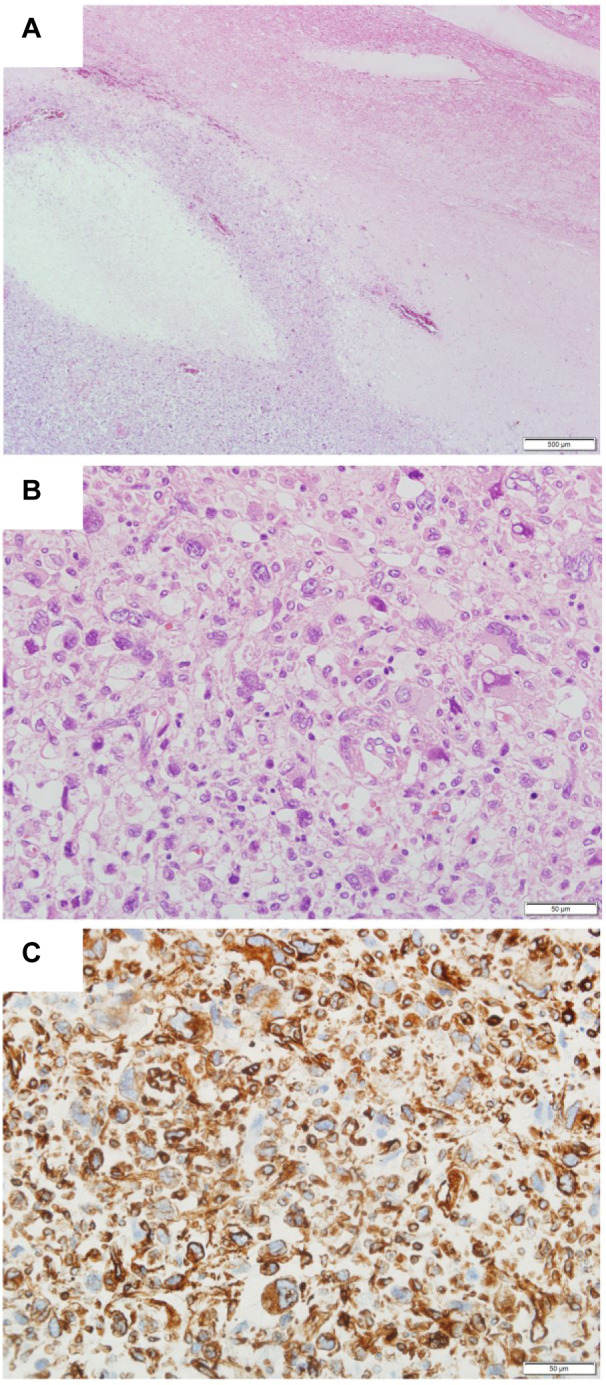
Microscopic examination of hematoxylin and eosin-stained slides of the tumor specimen showed no pattern or any specific tissue architecture at low magnification (A), indicating an undifferentiated malignant tumor. In addition, highly deformed nuclei were found on high magnification (B). Immunostaining was positive for vimentin (C).

A CT-guided needle biopsy of the mediastinal tumor revealed a malignant nonepithelial tumor of unknown origin. Because the patient presented with respiratory failure in poor clinical condition, she received palliative care without any active treatment. Her health gradually deteriorated, and she died 33 days after admission. Post-mortem pathological examination was performed with the consent of her family; a mediastinal tumor containing a large amount of nonserous fluid that invaded the heart and large blood vessels was macroscopically found. Microscopic examination of hematoxylin and eosin-stained slides of the tumor specimen showed no pattern or any specific tissue architecture at low magnification (Figure 3A(Fig. 3)), indicating an undifferentiated malignant tumor. In addition, highly deformed nuclei were found on high magnification (Figure 3B(Fig. 3)). Immunostaining showed positive results for vimentin (Figure 3C(Fig. 3)) and negative results for CD5, CD31, CD34, CD45, CD68, CD138, CK-CAM5.2, CK-AE1/1, AE3, EMA, calretinin, desmin, α-SMA, MA-HHF, S100, D2-40, HMB45, EMA, and MelanA. Thus, the condition was diagnosed as undifferentiated pleomorphic sarcoma.

## Discussion

Various tumors originate in the mediastinum. In Japan, the incidence of thymoma is highest (39.8 %), followed by congenital cysts (20.4 %), and neurogenic tumors (10.7 %). Thymus cancer accounts for 5.8 %, and germ cell tumors account for 5.1 % (Masuda et al., 2015[3]). Undifferentiated pleomorphic sarcoma belongs to a group of histologically undifferentiated sarcomas containing spindle shaped, circular, polygonal, and several atypical giant cells. Undifferentiated pleomorphic sarcoma, previously known as malignant fibrous histiocytoma (MFH), is the most common soft tissue sarcoma according to the World Health Organization (WHO) diagnostic criteria for soft tissue sarcoma, which was revised in 2013. Undifferentiated pleomorphic sarcoma is now classified as an independent histotype (Fletcher et al., 2013[1]). According to previous studies, MFH often develops in the extremities, retroperitoneum, and abdominal cavity but rarely in the chest (Shibuya et al., 1991[8]; Weiss et al., 1978[9]). Murakawa et al. have summarized 35 mediastinal cases of MFH (Murakawa et al., 2001[4]). Thereafter, several other cases of undifferentiated pleomorphic sarcoma have been reported (Nakamura et al., 2019[5]). Sarcoma in the mediastinum, particularly undifferentiated pleomorphic sarcoma, is rare.

There is no standard therapy for the treatment of undifferentiated pleomorphic sarcoma other than surgery. A previous study reported the case of a patient who underwent surgery for pleomorphic sarcoma in the mediastinum and survived for > 1 year after surgery (Kitano et al., 2010[2]). However, in another study, surgery was performed in a patient with pleomorphic sarcoma, but there was recurrence with gastrointestinal metastasis 3 months postoperatively. The patient died 4 months later (Okuda et al., 2015[7]). The effects of radiotherapy and chemotherapy are not defined (Nakamura et al., 2019[5]). Some reports have shown that adriamycin and ifosfamide are effective against MFH (Nishida et al., 2011[6]). In the present case, surgery was not indicated because her tumor had already invaded the pericardium at the time of admission. We believed that chemotherapy was not a treatment option because the tumor had compressed the superior vena cava and the patient presented with severe heart failure; the cardiotoxic effects of adriamycin were not likely to have been tolerated by the patient.

Treatment in this type of patient is challenging because tumor is often diagnosed at an advanced stage or after recurrence (Nakamura et al., 2019[5]; Okuda et al., 2015[7]). Undifferentiated pleomorphic sarcomas may have a rapidly progressive course. Therefore, clinicians should be cautious with the differential diagnosis and treatment of this disease. Because of the revised version of the WHO diagnostic criteria for soft tissue sarcoma in 2013, distinction between undifferentiated pleomorphic sarcoma and MFH has become challenging. Currently, the reports of mediastinal undifferentiated pleomorphic sarcoma are limited. Thus, further collection and analysis of such cases is required. 

We experienced a case of undifferentiated pleomorphic sarcoma that developed in the anterior mediastinum and was diagnosed by postmortem pathological autopsy. We need to recognize that there is a case in which the disease progresses rapidly as in this case and a diagnosis cannot be made. It is important to consider undifferentiated pleomorphic sarcoma as a differential diagnosis of anterior mediastinal tumor, and histological examination for diagnosis should be actively performed and early treatment should be considered.

## Acknowledgements

The authors would like to thank Enago (www.enago.jp) for the English language review. The authors would like to thank Mitsunori Yamakawa and Yuka Urano for the comments of pathological findings.

## Conflict of interest

We have no financial relationship with the organization that sponsored the research.
